# The aryl hydrocarbon receptor is required for induction of p21^cip1/waf1^ expression and growth inhibition by SU5416 in hepatoma cells

**DOI:** 10.18632/oncotarget.16056

**Published:** 2017-03-09

**Authors:** Edmond F. O’Donnell, Hyo Sang Jang, Martin Pearce, Nancy I Kerkvliet, Siva Kumar Kolluri

**Affiliations:** ^1^ Cancer Research Laboratory, Oregon State University, Corvallis, Oregon, USA; ^2^ Department of Environmental and Molecular Toxicology, and Environmental Health Sciences Center, Oregon State University, Corvallis, Oregon, USA

**Keywords:** AhR, proliferation, ligands, nuclear receptor, Arnt

## Abstract

The aryl hydrocarbon receptor (AhR) is a potential clinical target for cancer and autoimmune dysfunction. Identifying selective AhR modulators that produce desirable clinical outcomes represents an opportunity for developing new anti-cancer agents. Repurposing clinically-used drugs with established safety profiles that activate the AhR represents a good starting place to pursue this goal. In this study, we characterized the AhR-dependent effects of SU5416 (Semaxanib) following its identification in a small-molecule library screen. SU5416 potently activated AhR-dependent reporter genes, induced AhR nuclear localization, facilitated AhR-DNA binding, and increased, expression of its endogenous target genes. SU5416 significantly inhibited proliferation of Hepa1 hepatoma cells in an AhR-dependent manner, but did not induce apoptosis. SU5416 also inhibited the growth of human HepG2 liver cancer cells. The effects of SU5416 correlated with an increased G1 population and increased expression of cell cycle inhibitor p21^cip1/waf1^ at both the mRNA and protein level. Increased expression of p21^cip1/waf1^ by SU5416 required expression of both AhR and Arnt. In addition, evidence for long-term activation of the AhR *in vivo* by a single dose of SU5416 was identified by analyzing published microarray data. Our results provide support for continued investigation of the AhR as therapeutic for cancers such as hepatocellular carcinoma. In addition, our findings raise the possibility that some of the previously observed anti-proliferative effects of SU5416 may be due to activation of the AhR.

## INTRODUCTION

The aryl hydrocarbon receptor (AhR) is a ligand activated transcription factor known primarily as the mediator of toxicity of dioxins and polyaromatic hydrocarbons, the most-well characterized of which is 2, 3, 7, 8-tetrachlorodibenzo-*p*-dioxin (TCDD) [1, 2]. The AhR is usually present in the cytosol bound to two HSP90 molecules and one molecule of XAP-2, also known as AIP [1, 3]. Activation of the AhR by a ligand results in dissociation of this complex, translocation to the nucleus, and formation of a heterodimeric complex with the AhR nuclear translocator (Arnt), also known as HIF-1β. Subsequent recruitment of transcriptional machinery by the AhR/Arnt complex modulates the expression of a number of genes, notably CYP1A1, which is involved in first pass metabolism and modulate the affinity of AhR-activating ligands towards the AhR [1].

Regulation of cellular proliferation is a key aspect of AhR biological function. TCDD and other AhR ligands have been shown to upregulate expression of the cyclin-dependent kinase (CDK) inhibitor p27^kip1^ resulting in G1 arrest and growth inhibition of hepatoma cells [4]. In a similar manner, the CDK inhibitor p21^cip1/waf1^ has also been shown to be a ligand-dependent AhR target gene in some cell types. Whereas TCDD does not increase expression of p21^cip1/waf1^ in human SK-N-SH neuronal cells [5], functional XREs/AhREs in the p21^waf1/cip1^ promoter have been identified that are responsive to 3-methylcholanthrene (3-MC) [6]. Regulation of microRNAs by the AhR has also recently been demonstrated, with implications for metastasis of breast cancer [7]. The ability of the AhR to function as a tumor suppressor in the absence of exogenous ligands has been demonstrated in a mouse model of prostate cancer [8], but this appears to be a context dependent effect, with some studies suggesting a pro-proliferative function of the AhR in cancer [9].

The anti-proliferative functions of the AhR and its ability to be activated by a spectrum of diversely structured small molecules make it an attractive therapeutic target [10]. However, harnessing the therapeutic potential of the AhR presents unique challenges due to the toxic endpoints observed with certain classes of AhR ligands such as dioxins and other environmental PAHs. To address this challenge, a number of recent studies have identified novel selective AhR modulators (SAhRMs) [10] or utilized drugs currently in use in the clinic and with a previously unappreciated capacity to activate the AhR, as candidates for anti-cancer therapeutics in breast cancer and melanoma [11, 12]. Especially, development of anti-cancer AhR ligands via repurposing of FDA approved drugs and drugs that have reached testing in clinical trials whose toxicity profiles have been well characterized represents an excellent opportunity to expedite development of the AhR as a clinical drug target by addressing concerns associated with dioxin-mediated AhR activation.

In the present study, we conducted a xenobiotic response element driven luciferase reporter-based small-molecular library screen and identified SU5416 (Semaxanib) as a putative AhR ligand. SU5416 was also recently described as an AhR ligand in the context of immune suppression following an independent screening effort [13]. Importantly, SU5416 has been characterized extensively as an inhibitor of the VEGFR tyrosine kinase Flk-1/KDR [14], and investigated in a number of clinical trials [15–18]. Indeed, SU5416 at one point progressed to phase III clinical trials for treatment of colorectal cancer in combination with other chemotherapeutics [19–22]. Envisioning a potential repurposing of this molecule via the AhR in a responsive cancer, we sought to characterize the AhR-dependent functional effects of SU5416, focusing on its anti-proliferative effects in hepatoma. Our results showed that SU5416 strongly activates the AhR and Arnt signaling, which mediates its potent anti-proliferative effects in hepatoma cells.

## RESULTS

### SU5416 activates aryl hydrocarbon receptor signaling

**Figure 1:**
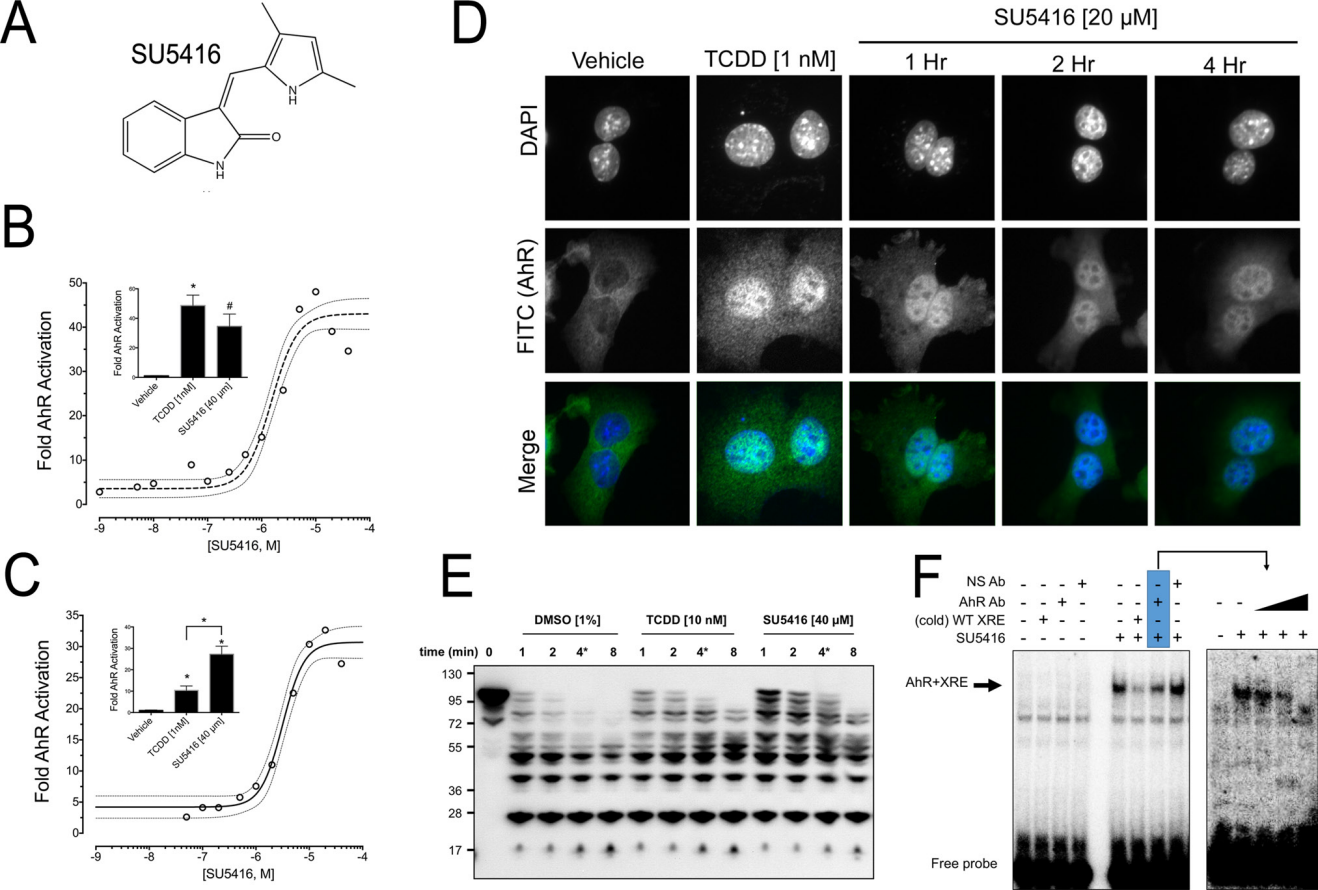
SU5416 activates the AhR (**A**) Structure of SU5416. (**B–C**) SU5416 increases AhR driven luciferase reporter gene expression in a dose dependent manner in mouse Hepa1 and human HepG2 cell lines, respectively. Data points are from at least three independent experiments, and a fitted non-linear regression is shown (dashed lines show the 95% CI). Inset figures shown comparison of maximum reporter activation with 1 nM TCDD and the highest concentration of SU5416 tested (40 μM). (Mean ± SEM, ^#^*p* < 0.01, *p < 0.001). (**D**) SU5416 induces AhR nuclear localization similar to TCDD. (**E**) SU5416 delays partial AhR proteolysis similar to TCDD. Hepa1 cell extracts were incubated with the indicated ligands or vehicle (DMSO), incubated with subtilisin, and analyzed by Western blot with a polyclonal N-terminal antibody to detect AhR cleavage products. Data are representative of at least three similar experiments. (**F**) The left panel shows EMSA performed with Hepa1 cell extracts showing formation of an AhR/XRE-probe complex in the presence of SU5416 (arrow), a non-labeled (cold) probe, 0.1 μg of AhR-antibody, or a non-specific antibody (Anti-p27). The right panel shows a similar experiment in which three different concentrations of AhR-antibody (left to right: 0.1 μg, 0.3 μg, and 1 μg) were added to the reactions.

A xenobiotic-response element (XRE) reporter-based screen of the LOPAC 1280 library was performed to identify small molecule activators of the AhR [23]. Through this screen, we identified SU5416 as a putative AhR activator (Figure 1A). We first wanted to characterize the ability of SU5416 to activate the AhR, and in addition determine the mechanism of this activation by asking whether SU5416 is an AhR ligand. We performed a reporter gene assay in Hepa1 cells treated overnight with concentrations of SU5416 ranging from 1 nM to 40 μm. Consistent with the results of our small molecule screen, SU5416 strongly activated the AhR-reporter gene in a dose-dependent manner (Figure 1B). We observed noticeable AhR reporter gene induction by SU5416 beginning at 100 nM, which achieved maximum activation of the reporter system at 10 μM. The apparent EC_50_ of AhR reporter gene activation by SU5416 in the Hepa1 cells was 1.53 μM (EC_50_ 95% CI 1.16 to 2.01 μM).

We next tested whether SU5416 activates AhR signaling in human cells. Thus, we performed a reporter gene assay in human HepG2 hepatocellular carcinoma cells using an XRE-driven reporter. We found that SU5416 activated the human AhR in a manner similar to that of mouse AhR. Appreciable AhR reporter gene induction was observed beginning at 100 nM, reaching maximal activation at 20 μM. The apparent EC_50_ of SU5416 in the HepG2 XRE-based reporter gene assay was 3.17 μM (Figure 1C, EC_50_ 95% CI 2.44 to 4.12 μM).

Our next goal was to establish the manner by which SU5416 activates AhR signaling. The AhR is typically localized in the cytosol, and binding of the AhR to a ligand results in nuclear translocation. Immunofluorescence staining showed that SU5416 induced nuclear translocation of the AhR in Hepa1 cells after 4 hours similar to the AhR-ligand TCDD (Figure 1D). In addition to nuclear translocation, we performed two sets of *in vitro* assays to gain evidence for an interaction between AhR and SU5416. First, we conducted a limited proteolytic digestion of AhR in the presence of the vehicle control, 1 nM TCDD, or SU5416. High concentrations of both TCDD (10 nM) and SU5416 (40 μM) relative to the respective EC_50_ values of the compounds were selected to ensure saturation under *in vitro* conditions. Digestion of whole cell extracts of Hepa1 cells by the protease subtilisin in the absence of an AhR ligand generated a broad range of fragments that were easily detected with a polyclonal AhR antibody generated from the N-terminus (residues 1–402) of the receptor. (Figure 1E). Treatment with 10 nM TCDD as a positive control resulted in a greater intensity of fragments between 95 and 55 kDa. The same pattern, albeit more intense, was observed for SU5416. Based on the ability of the well-known AhR ligand TCDD to delay proteolysis, the similar pattern for SU5416 was taken as indirect but strong evidence that SU5416 binds to the AhR.

To obtain additional evidence that SU5416 is an AhR ligand, we performed an electrophoretic mobility shift assay (EMSA) with Hepa1 whole cell extracts in the absence or presence of SU5416. Whereas there was no increase of ^32^P-labeled XRE-probe shift in the absence of SU5416, a significant increase in binding was noted in the presence of the compound. In support of the specificity of this interaction, addition of a cold XRE probe diminished the SU5416-induced probe shift. Likewise, addition of an AhR-antibody also reduced binding compared to a non-specific (anti-p27) antibody (Figure 1F, left panel). The effect of the AhR antibody was repeated using three different concentrations of antibody, and there was a dose-dependent decrease in SU5416-induced XRE-probe shift with increasing antibody amounts (Figure 1F, right panel). The collective results of the reporter gene assays, immunofluorescence studies, proteolysis studies, and EMSA all supported that SU5416 is an AhR activator, and does so by binding the receptor.

**Figure 2:**
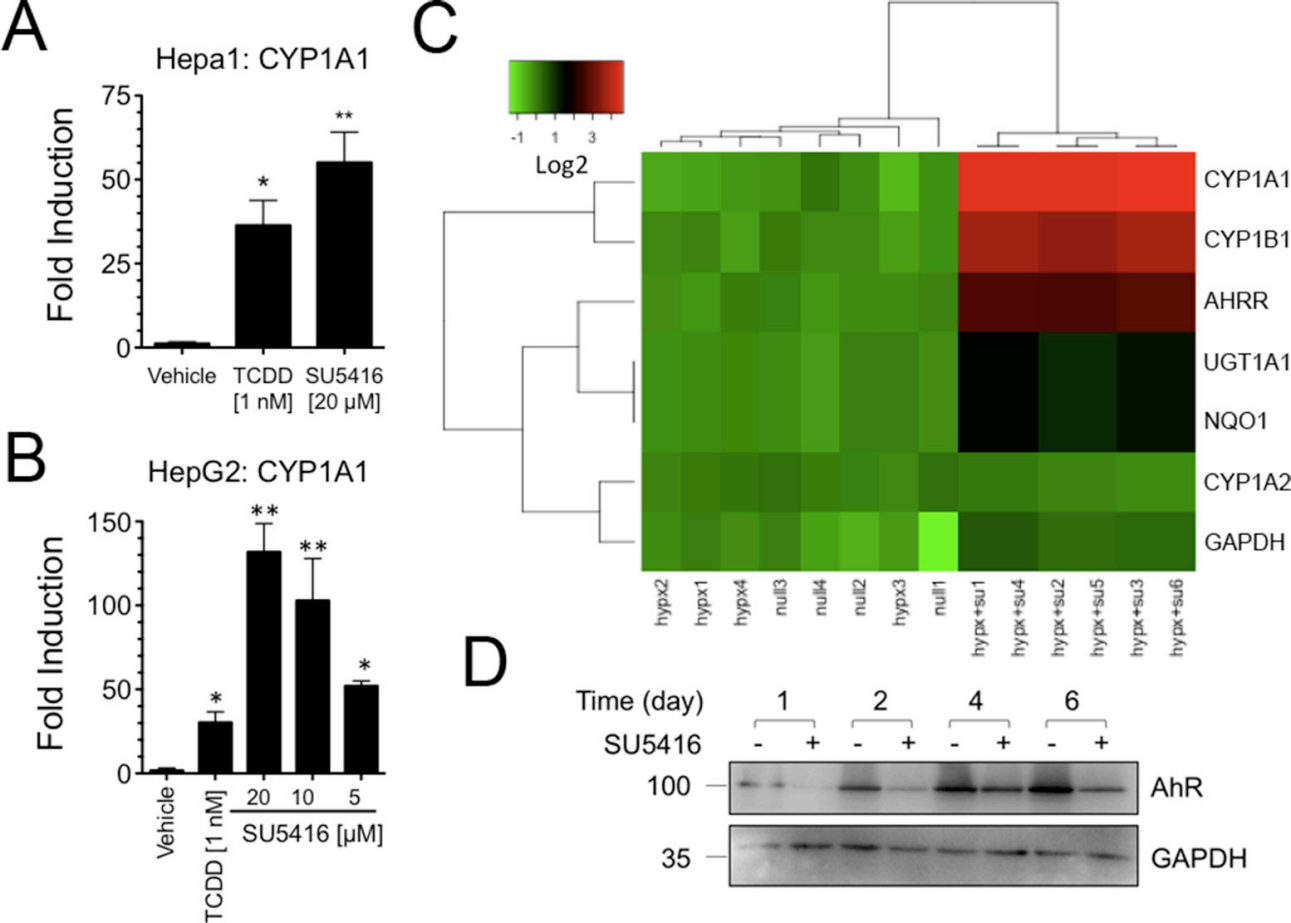
SU5416 increases the expression of AhR target genes (**A–B**) SU5416 activates the AhR target gene CYP1A1 in mouse and human hepatoma cells lines, respectively. Data are the mean ± SEM of three or more independent determinations. *p < 0.01, **p < 0.001. (**C**) Identification of an AhR gene induction signature *in vivo* form the lungs of rats treated with a single dose of SU5416. Intensity data is shown on a log2 intensity scale. Groups (bottom labels) consists of normoxia exposed rats (null), hypoxia exposed rats (hypx), and hypoxia exposed rats treated with SU5416 (hypx+SU); each column corresponds to an independent animal. (**D**) SU5416 suppresses AhR protein levels. Hepa1 cells were plated in 6 well plates and treated with SU5416 (20 μM). Cells were collected at the indicated time points, and whole cell lysates were prepared and analyzed by Western blot with the indicated antibodies.

Having determined that SU5416 activates the AhR through binding, we next asked if SU5416 is capable of regulating the expression of classic AhR target genes. Mouse Hepa1 cells were incubated overnight with TCDD or SU5416 and analyzed for expression of CYP1A1 (Figure 2A). As expected, 1 nM TCDD significantly increased the abundance of CYP1A1 by 36.4 ± 7.4 fold vs vehicle (*p* < 0.05; mean ± SEM, *N* = 3), while 20 μM SU5416 caused a fold increase of 55.1 ± 9.0 (*P* < 0.01 vs vehicle, *P* > 0.05 vs TCDD). A similar dose-dependent relationship between CYP1A1 activation and SU5416 concentration was observed for HepG2 cells (Figure 2B).

SU5416 has undergone extensive pre-clinical and clinical testing, and we were next interested if data from previous studies could be re-analyzed to detect AhR activation. To address this question, we searched the GEO database for *in vivo* gene expression experiments conducted with SU5416. We identified one such dataset in GEO in which Dahl/Salt-sensitive rats, a hypertension model, were exposed to SU5416 [24]. Specifically, Moreno-Vinasco *et al*. used three animal groups exposed to normoxia, hypoxia (10% inspired O_2_), or hypoxia with a single injection of 20 mg/kg SU5416 given at the onset of the study [25]. In their study, RNA was extracted from the lungs of animals 3.5 weeks after the single injection and analyzed using GeneChip Rat Genome 230 2.0 Arrays. Re-analysis of this curated data was performed with unsupervised hierarchical clustering of the treatment groups against a set of manually selected AhR target genes. We included the comparison with untreated hypoxia and normoxia to confirm that the observed changes in gene expression were due to SU5416 itself, rather than an effect of hypoxia. Despite being given only a single injection of SU5416, a dramatic AhR-gene signature was observed in rat lung after 3.5 weeks (Figure 2C), with significant upregulation of CYP1A1, CYP1B1, AHRR, NQO1, and UGT1A1 (> 2 fold, *p* < 0.05). Importantly, this set of genes clustered together in the hypoxia and normoxia groups, suggesting that it was due to SU5416 treatment and not a combinatorial effect.

The microarray data described above suggested that SU5416 can maintain AhR-activation over a protracted time. We determined if SU5416 could maintain a similar long-term activation of the AhR in cells in culture, with a onetime treatment. The AhR is rapidly degraded in the presence of ligand [26]. Thus, we reasoned that if SU5416 is stable and sufficiently potent, we might see continued downregulation of the AhR, suggesting continuous AhR activation by the compound. Hepa1 cells were treated for up to 6 days with SU5416, and at all time points analyzed (1, 2, 4, and 6 days), we noted significant downregulation of the AhR (Figure 2D).

### SU5416 inhibits Hepa1 cell proliferation but does not cause cell death

SU5416 is an experimental stage cancer therapeutic that has been tested in humans in clinical trials. Having demonstrated that SU5416 activates the AhR (Figures 1 and 2), we were next interested in the phenotypic consequences of the drug activation of the AhR. Specifically, we were curious if any of the effects of this molecule could be attributed to its ability to activate the AhR.

**Figure 3:**
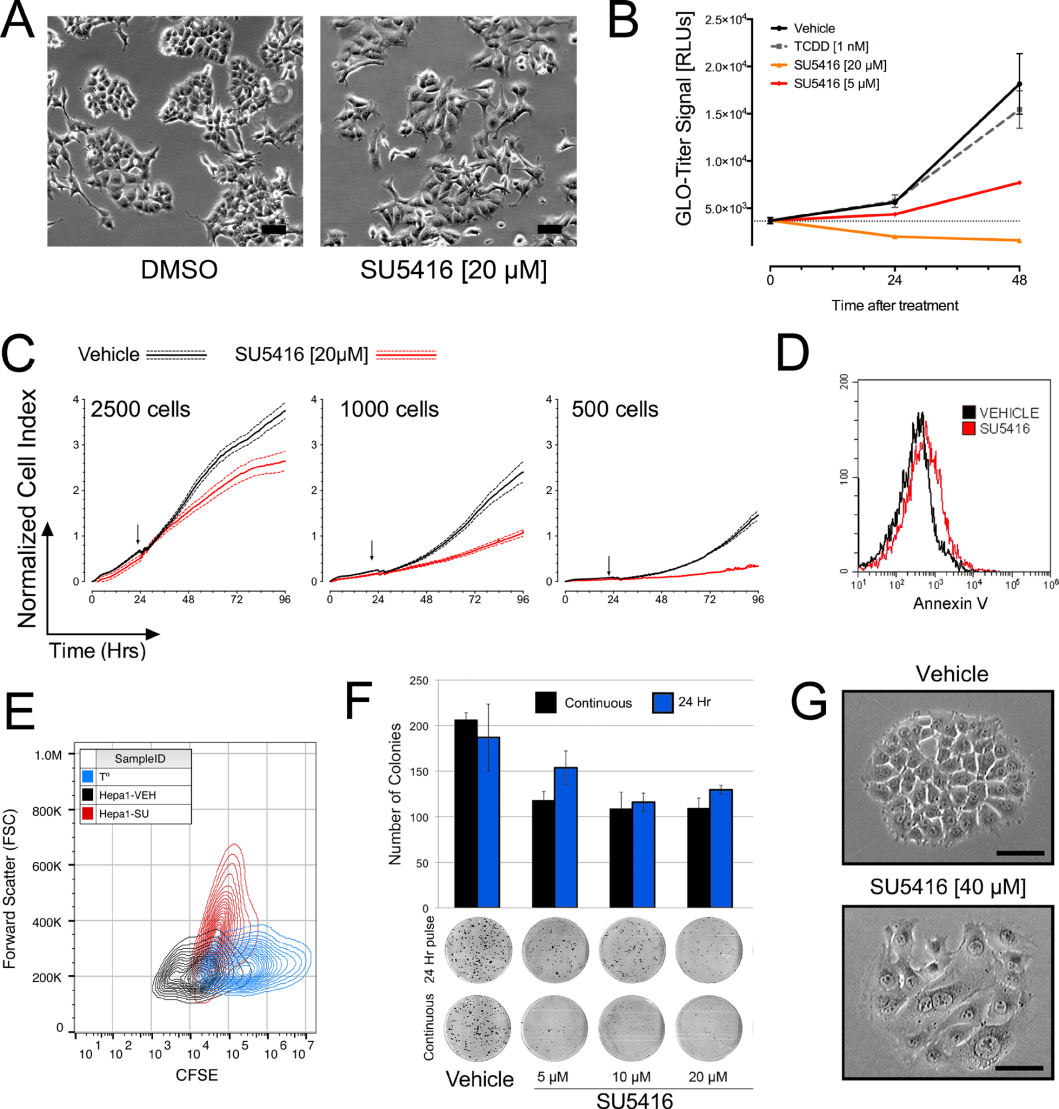
SU5416 inhibits the growth of hepatoma cells and induces a change in cellular morphology (**A**) Microscopy images of Hepa1 cells treated with DMSO or SU5416 for 24 hours. (**B**). Treatment with SU5416 inhibits the total number of Hepa1 cells in culture in a dose and time dependent manner. Cells were plated, grown overnight, and some wells were assayed with the Glo-Titer reagent fifteen minutes prior to the addition of treatment (Time 0). Plates were then assayed at 24 and 48 hours after treatment under the same conditions, and the data were compiled using the raw integrated signal (1 second) from the same luminometer. In this way, the data across time points could be compared to interrogate changes in signal reflecting cell proliferation. Data are the mean ± SEM, *n* = 4. (**C**) xCelligence assays of Hepa1 cells treated with vehicle or SU5416 (20 μM) at different initial plating densities as indicated. Cells were plated, grown for approximately 24 hours, and treated by the gentle addition of a concentrated stock solution of DMSO or SU5416 (vertical arrows indicate addition of treatment). Data were normalized according to cell concentration at the time of treatment and are thus comparable across all three panels. Data are the mean ± SEM of at least 4 biological replicates. (**D**) Annexin V staining of Hepa1 cells treated overnight with vehicle or SU5416 [20 μM]. (**E**). CFSE staining of Hepa1 cells treated for 48 hours with vehicle or SU5416 [20 μM]. Data are shown as a 2D-histogram with CFSE (FL1) vs forward scatter (FSC). T^O^ indicates the profile of cells stained at the beginning of the experiment, and decreasing fluorescence along the x-axis is indicative of dividing cells. (**F**) Colony formation assay in Hepa1 cells treated with SU5416 at the indicated concentrations either continuously or for 24 hours followed by replacement with fresh media. (**G**) Phase contrast microscopy images of Hepa1 cell colonies formed after approximately 72 hours under continuous treatment conditions. Bars represent equivalent image size within a given panel.

We began our investigation of the consequences of AhR activation by SU5416 with basic microscopy. Exponentially growing Hepa1 cells in 6 well-plates were treated with vehicle, 1 nM TCDD, or SU5416 overnight. For this analysis, the concentrations of SU5416 were selected based on the dose-response activation noted in Figure 1, ranging from 20 μM to 1 μM, representing activation-saturating conditions and low-activation conditions, respectively. After overnight incubation, we made several general observations based on cells treated with 20 μM SU5416 (Figure 3A). First, the total number of cells were less than vehicle treated cells. Secondly, the morphology of SU5416 treated cells was altered, with the cells exhibiting an engorged appearance. We noted an absence of obvious indicators of cell death such as floating cells in the culture dish and membrane blebbing.

We next quantitatively evaluated the effects of SU5416 on Hepa1 cells using a viability assay (Promega Glo-Titer) that quantifies cell abundance according to total ATP content. For this assay, Hepa1 cells seeded at an initial density of 1000 cells/well in microtiter plates were grown overnight, after which a subset of wells were assessed by Glo-Titer to obtain a baseline reading, while the remainder were treated with vehicle, 1 nM TCDD, or SU5416 at two different concentrations. Quantification of vehicle treated cells 48 hours later revealed a 4.9-fold increase in Glo-Titer signal, consistent with several doublings of cells as expected (Figure 3B). Cells treated with TCDD exhibited a similar growth as vehicle. Treatment of Hepa1 cells with 5 μM, 20 μM SU5416 for 24 and 48 hours led to strong decrease in Glo-Titer signals.

Our observation of altered cellular morphology (Figure 3A) and decreased cellular abundance (Figure 3B) suggested to us that SU5416 may induce a growth-inhibitory effect rather than a cytotoxic effect. To address this possibility, we next evaluated the effect of SU5416 on Hepa1 cells using the xCelligence assay, which generates a real-time ‘cell index’ measurement that collectively represents cell proliferation/abundance, adhesion, and morphology based on impendence caused by cells over small electrodes in specialized microtiter plates. Three different densities of cells were tested (Figure 3C), and in each experiment, treatment of Hepa1 cells with SU5416 decreased the normalized cell index compared with vehicle treated cells, but not below that of the value at the start of the experiment, suggesting that the cells were not proliferating, but remained viable. Conversely, a cytotoxic effect could be expected to produce in a decrease in cell-index below that of the value at the start of treatment. To further rule out the possibility that SU5416 induces cell death in Hepa1 cells, we performed an apoptosis assay with Annexin V. Consistent with our data thus far, SU5416 did not induce a significant increase in Annexin V staining of Hepa1 cells incubated with the compound (Figure 3D). We confirmed that SU5416 does not induce apoptosis in Hepa1 cells by evaluating nuclear morphology with DAPI staining (Supplementary Figure 1). Conversely, when we stained Hepa1 cells with CFSE and incubated the cells with either vehicle or SU5416 for 48 hours, we found that the CFSE signal was much more diminished in vehicle treated cells compared with those treated with SU5416 (Figure 3E). As CFSE is diluted during cellular division, this result suggested a growth-inhibitory effect of SU5416, consistent with our xCelligence data described above. Furthermore, when plotted against the forward-scatter (FSC) parameter, which can be considered a measurement of cell size, SU5416 treatment dramatically increased FSC compared to vehicle treatment. Lastly, DNA-profile analysis of Hepa1 cells treated long-term (72 hrs) with SU5416 showed no evidence of increased ploidy in the cells (data not shown).

To further study the effect of SU5416 on Hepa1 cells, we next performed colony formation assays. Two treatment schemes for SU5416 were utilized: continuous treatment and a short-duration treatment, in which the SU5416-containing media was replaced with fresh media after 24 hours. In both treatment methods, the number of colonies that reached a sufficient size to be counted was decreased in a dose-dependent manner by SU5416. However, careful inspection of the plates revealed numerous punctate colonies (Figure 3F, lower panel). These smaller colonies were much more apparent in the pulse-treatment group compared with the continuous treatment group, suggesting some degree of recovery by the cells after removal of SU5416. Interestingly, imaging of cells during growth under colony forming conditions provided further evidence of the effect of SU5416 on cell morphology on size. In the two representative colonies initiated by a single cell, despite covering approximately the same surface area, a dramatic difference in the number of cells within the colony was noted (Figure 3G).

### AhR is required for the growth-inhibitory effects of SU5416

**Figure 4:**
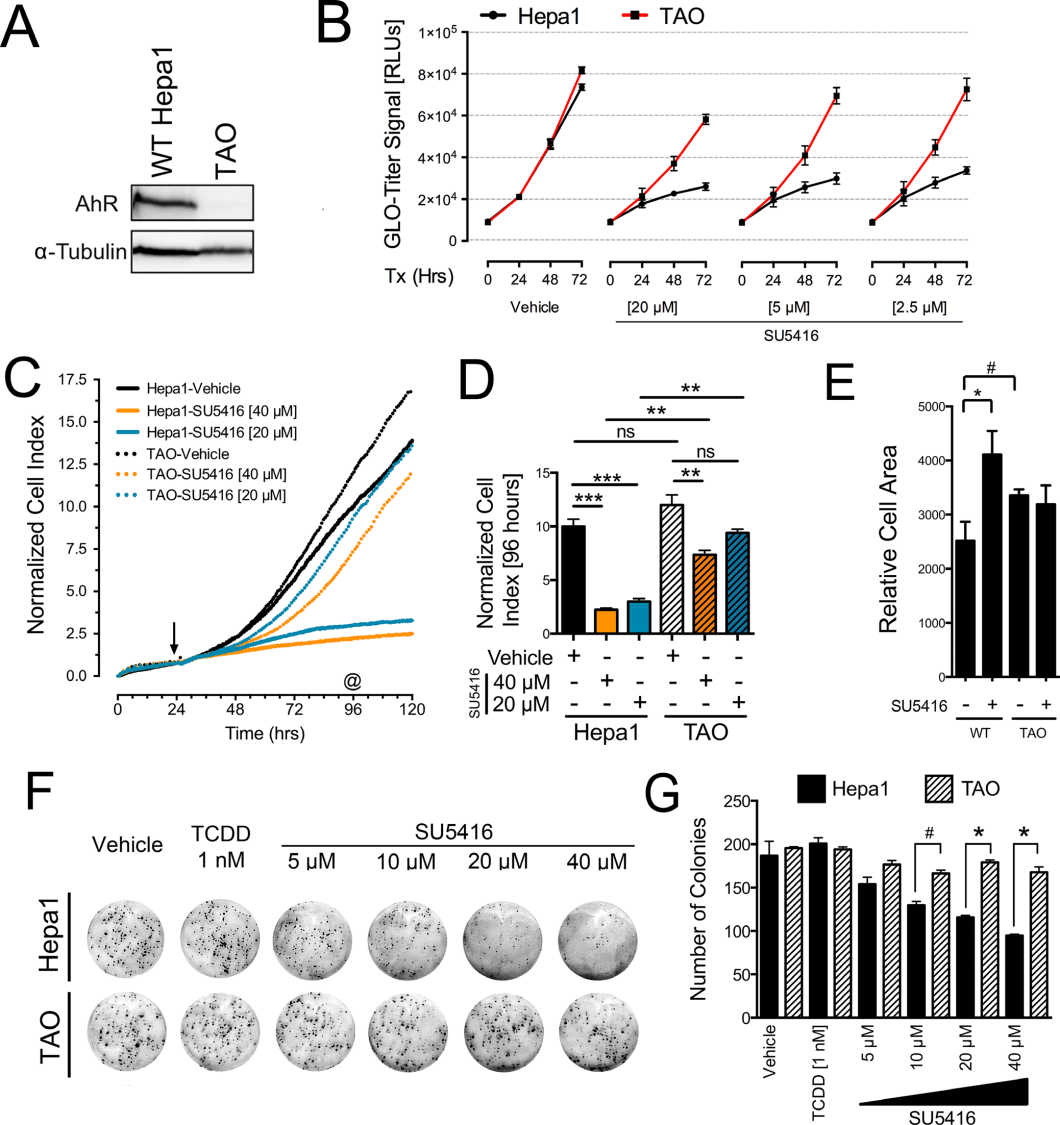
The anti-proliferative effects of SU5416 in Hepa1 cells are AhR-dependent (**A**) Western blot depicting the relative AhR abundance in Hepa1 and TAO cells. (**B**) Cell-Titer Glo assays with Hepa1 and TAO cells treated with the indicated concentrations of SU5416 for 72 hrs. The data were analyzed as described for Figure 3B, and the represent the mean ± SEM, *n* = 3. Data are representative of three independent experiments. (**C**) xCelligence real time proliferation analysis of Hepa1 and TAO cells (initial seeding density 1000 cells/ well). Initiation of treatments is depicted by an arrow. (**D**) ANOVA analysis of the 96 hr time point (72 hr treatment, indicated by as in C); **p < 0.01, ***p < 0.001, *n* = 2. (**E**) Cell area of Hepa1 and TAO cells treated with vehicle or SU5416 [40 μm] for 48 hrs. Bars indicate the mean ± SEM surface area of cells obtained from five representative images, and are expressed as relative cell area (arbitrary units^2^); ^#^*p* < 0.05, *p < 0.01. (**F**) Colony formation assay showing the sensitivity of Hepa1 cells vs Tao cells to SU5416. Cells were treated for 24 hours with vehicle, TCDD, or SU5416 at the indicated concentrations, after which the media was replaced with normal cell culture media (**G**) ANOVA analysis of colony counts, ^#^*p* < 0.05, *p < 0.01.

The concentrations of SU5416 that facilitated growth inhibition of Hepa1 cells (Figure 3) were consistent with those necessary for AhR activation (Figures 1, 2). To determine if the growth inhibitory effects of SU5416 observed above were mediated by the AhR, we turned to a cell culture model of differential AhR expression. We first used Hepa1 cells and the derivative cell line TAO, which has a significantly lower abundance of AhR (Figure 4A), which we have used previously to characterize the AhR-dependent anti-proliferative effects of raloxifene [27]. First, we used the Glo-titer viability assay to quantify the time- and dose-dependent effects of SU5416 in the presence of high or very low levels of AhR (Figure 4B). SU5416 at three concentrations of 20, 5, and 2.5 μM significantly inhibited the growth of Hepa1 cells compared to TAO cells (Figure 4B).

To confirm these effects, we utilized the xCelligence assay to monitor cell growth in real time as a function of AhR status. Based on our experience with Hepa1 cells treated with SU5416 at different initial densities, we performed the assay with a low starting cell density to specifically focus on the growth-inhibitory effects of SU5416 according to AhR expression. The normalized cell index of Hepa1 cells treated with SU5416 was significantly less than that of vehicle treated cells, whereas the normalized cell index for TAO cells was decreased far less by SU5416 treatment (Figure 4C). We quantified the xCelligence data by performing ANOVA on a snapshot of the normalized cell indices at 96 hours, which was after 72 hours of treatment (Figure 4D). SU5416 at 40 μM and 20 μM significantly decreased the normalized cell index by 78% and 70% vs vehicle treated cells, respectively (*P* < 0.001 for both). Conversely, TAO cells treated with SU5416 at 40 and 20 μM were inhibited by only 39% (*P* < 0.01) and 22% (*P* > 0.05), respectively. Thus, SU5416 dramatically inhibited the proliferation of Hepa1 cells while TAO cells were not significantly affected.

In Figure 3 we noted that SU5416 altered the morphology of Hepa1 cells, resulting in an enlarged cell appearance. To quantify this effect and determine whether it is dependent on AhR-expression, we took representative microscopy images of vehicle or SU5416 treated Hepa1 or Tao cells. After manually counting the number of cells in five of these images per group, we performed thresholding to define the relative areas occupied by cells. By dividing the number of cells with the area of the image occupied by cells, we were able to approximate mean cell surface area. Consistent with our observations thus far (Figure 3), SU5416 significantly increased the relative cell surface area of Hepa1 cells (*p* < 0.0001). In addition, while TAO cells had a somewhat larger cell surface area compared with Hepa1 cells (*p* < 0.01), this profile was not significantly altered by treatment with SU5416 (Figure 4E). Also consistent with our data thus far, SU5416 decreased the colony formation of Hepa1 cells but not TAO cells, while TCDD had no effect on colony formation (Figures 4F–4G).

### Arnt is required for the growth-inhibitory effects of SU5416 in Hepa1 cells

**Figure 5:**
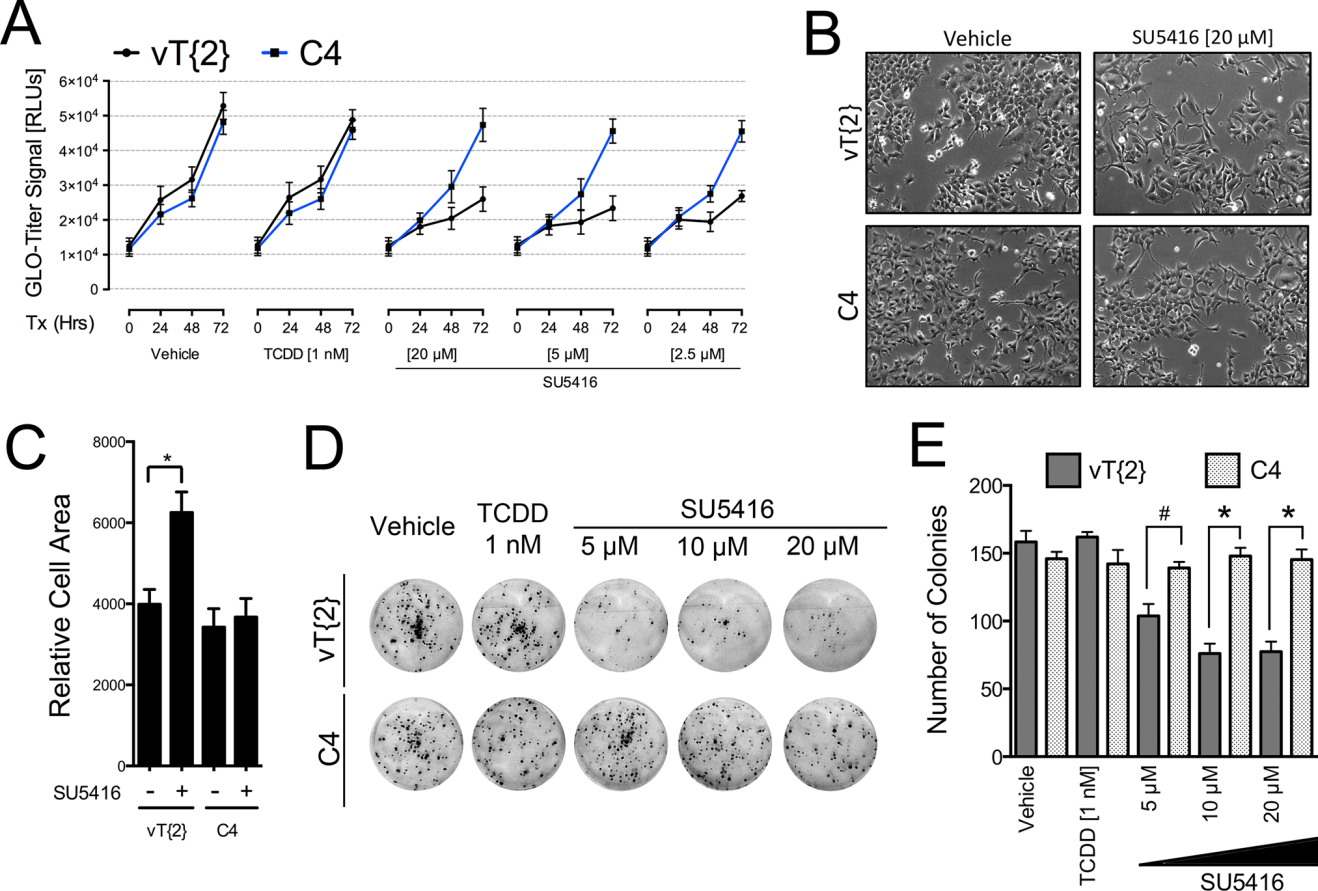
Arnt is required for the anti-proliferative effects of SU5416 in Hepa1 cells (**A**) Promega Glo-Titer assay with C4 (Arnt deficient) and vT{2} (rescued Arnt expression) cells treated with varying doses of SU5416 for 72 hours and analyzed as described for Figure 3B. Data are the mean ± SD, *n* = 3, and are representative of three independent experiments. (**B**) Microscopy images of C4 and vT{2} cells treated with vehicle or SU5416 (20 μM) (**C**). Cell area of C4 and vT{2} cells treated with vehicle or SU5416 [40 μm] for 48 hrs analyzed as described in Figure 4E. Bars indicate the mean ± SEM surface area of a single cell, expressed as relative cell area (arbitrary units^2^), generated from phase contrast microscopy images of 5 h.p. fields); *p < 0.01. (**D**) Colony formation assay showing the sensitivity of C4 and vT{2} cells to SU5416. Cells were treated with vehicle, TCDD, or SU5416 at the indicated concentrations for the duration of the assay (continuous treatment). (**E**) ANOVA analysis of colony counts, ^#^*p* < 0.05, *p < 0.01.

The AhR nuclear translocator (Arnt) is the obligatory heterodimer of the AhR and is required for AhR-mediated gene activation. Therefore, we next asked whether Arnt is required for the anti-proliferative effects of SU5416. We used C4 and vT{2} cell lines, which express a mutated, transcriptionally inactive Arnt and re-expressed WT Arnt protein, respectively [23]. Similar to our observation for Hepa1/TAO cells (Figure 4B), absence or presence of Arnt activity had no effect on cell proliferation, TCDD treatment had no effect on these cells (Figure 5A). On the other hand, SU5416 inhibited the growth of vT{2} cells in a dose and time-dependent manner, while C4 cells were largely unaffected (Figure 5A). We assessed the effect of SU5416 on C4 and vT{2} cells visually, and again noted the same enlarged appearance of SU5416-treated vT{2} cells, but not C4 cells (Figure 5B). Similar to the results shown in Figure 4, treatment with SU5416 increased cell surface area only in the presence of functional Arnt (Figure 5C). Lastly, we tested the effects of SU5416 on vT{2} and C4 cells in colony forming assays. We found that vT{2} cells were more sensitive to SU5416 than C4 cells during continuous exposure to SU5416 (Figure 5D–5E).

### SU5416 upregulates CDK inhibitor p21^waf1/cip1^ in an AhR- and Arnt-dependent manner

**Figure 6:**
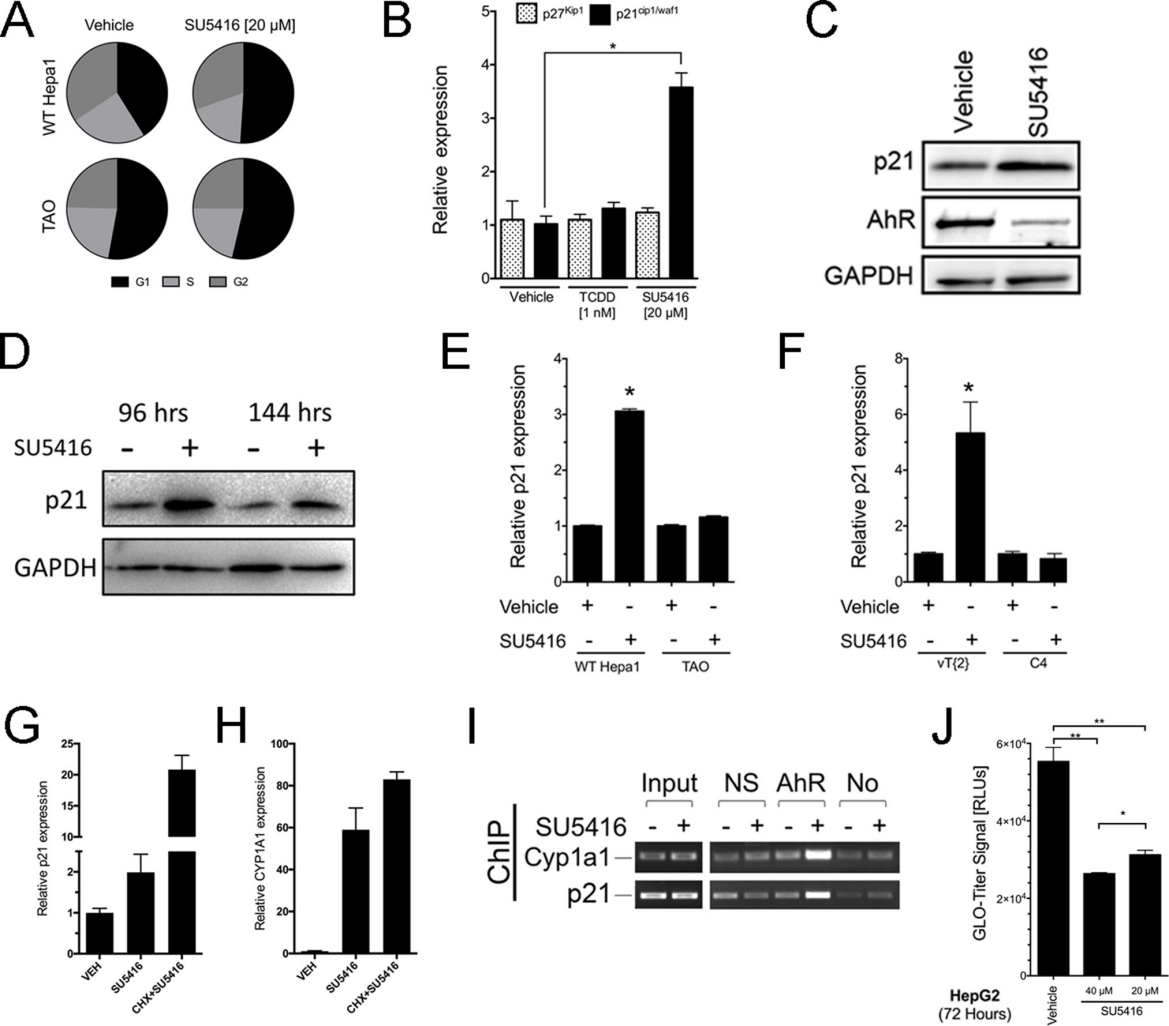
SU5416 increases the population of cells in the G1 phase and directly upregulates CDK inhibitor p21waf1/cip1 in an AhR- and Arnt-dependent manner (**A**) Cell cycle analysis of Hepa1 and TAO cells treated with vehicle (0.1% DMSO) or SU5416 for 48 hours, represented as parts of a whole graphs (*n* = 5). (**B**) qPCR analysis of p27^Kip1^ and p21^waf1/cip1^ expression following treatment with vehicle, TCDD, or SU5416 for 24 hours in Hepa1 cells. Data are the mean ± SEM of three independent determinations (*p* < 0.05 vs vehicle). (**C–D**) Western blot for p21 expression in Hepa1 cells treated with vehicle or SU5416 (20 μM) for 24 hours (**C**), or longer time points of 96 and 144 hours (D). GAPDH was included as a protein loading control. (**E–F**) Analysis of p21 expression by qPCR in (E) WT Hepa1 and TAO and (F) C4 and vT{2} cells, respectively, treated with SU5416 (20 μM) for 24 hours (Mean ± SEM, *n* = 3 *p < 0.0001 vs respective vehicle). (**G–H**) Hepa1 cells were treated with SU5416 in the absence or presence of cycloheximide for 18 hours and the respective expression of (G) p21^waf1/cip1^ and (H) CYP1A1 were analyzed. Cycloheximide resulted in a super-induction, rather than suppression, of gene expression. (**I)** Hepa1 cells were treated with SU5416 and analyzed by chromatin immunoprecipitation (ChIP) with an AhR antibody. NS, non-specific antibody; AhR, AhR antibody; No, no antibody. (**J**) HepG2 cells were plated in 96 well plates (2000 cells/well) and grown overnight. The indicated treatments were then added, and the cells were analyzed after 72 hours with Glo-Titer reagent. Data are the mean ± SD, *n* = 4. **p < 0.0001, *p < 0.05.

We next investigated the mechanism of SU5416-induced growth inhibition downstream of the AhR activation. Previous studies have shown that certain ligands of the AhR such as TCDD and 3-methylchloranthrene can increase the expression of the CDK inhibitors p27^kip1^ and p21^waf1/cip1^ and induce G1 arrest [4, 6, 28, 29]. Cell cycle analysis of WT Hepa1 and TAO cells treated with SU5416 revealed that SU5416 treatment increased the G1 population of Hepa1 cells while the percentage of TAO cells in the G1 phase between vehicle and SU5416 was essentially unchanged (Figure 6A). Specifically, SU5416 increased the percentage vehicle treated Hepa1 cells in the G1 phase from 40.6 ± 4.4% to 51.2 ± 3.3%, (*p* < 0.05, *n* = 5), whereas the difference between vehicle and SU5416 treated TAO cells was insignificant (*p* > 0.05, *n* = 5). We next asked whether p27^kip1^ and/or p21^waf1/cip1^ were upregulated by SU5416. The mRNA levels of p21^waf1/cip1^, but not p27^kip1^, were significantly upregulated by SU5416 (Figure 6B). We confirmed the upregulation of p21^waf1/cip1^ by SU5416 at the protein level. In addition, we observed AhR receptor downregulation by SU5416 (Figure 6C) at 24 hours, consistent with our previous observation (Figure 2D) and the literature [26]. We also tested whether SU5416-induced p21^cip1/waf1^ expression was maintained after relatively longer treatment intervals, and found that p21^cip1/waf1^ induction was maintained even after 96 and 144 hours of treatment (Figure 6D). Finally, we confirmed that the transcriptional induction of p21^waf1/cip1^ by SU5416 was dependent on both the AhR and Arnt expression. The expression of p21^waf1/cip1^ was induced by SU5416 in AhR expressing Hepa-1 cells, but not in TAO cells (Figure 6E). Similarly, p21^waf1/cip1^ was induced in functional Arnt expressing vT{2} cells, but not in C4 cells (Figure 6F).

### SU5416 activates p21^waf1/cip1^ directly through the AhR

We showed that expression of p21^waf1/cip1^ is induced by SU5416 only in the presence of functional AhR signaling (Figures 6E–6F). In addition, previous studies have described a role of the AhR as a transcriptional regulator of p21^waf1/cip1^ [6, 30]. Thus, it seemed likely that p21^waf1/cip1^ would be a direct transcriptional target of AhR activated by SU5416. To test this possibility, we treated Hepa1 cells with SU5416 in the presence or absence of cycloheximide, a protein synthesis inhibitor. If upregulation of p21^waf1/cip1^ by SU5416 is inhibited in the presence of cycloheximide, it would suggest that induction of an intermediary gene product is involved, whereas a lack of such an inhibition would argue for direct activation via the AhR. Consistent with the latter scenario, both p21^waf1/cip1^ and CYP1A1 mRNA expression were not inhibited upon co-treatment with cycloheximide (Figure 6G–6H). Instead, the expression of both genes was increased in the presence of cycloheximide, a phenomenon that has been reported previously for the AhR target gene CYP1A1 [31, 32]. To confirm that the AhR directly activates p21^waf1/cip1^ through a transcriptional mechanism, we performed chromatin-immunoprecipitation (ChIP) experiments. Recruitment of the AhR to p21^waf1/cip1^ and CYP1A1 promoters was observed only in cells treated with SU5416 and immunoprecipitated with an AhR antibody (Figure 6I), but not after immunoprecipitation with a non-specific antibody or no-antibody control.

### SU5416 inhibits proliferation of human HepG2 cells

To extend our findings of the growth inhibitory effects of SU5416 in mouse Hepa1 cells, we utilized the human HepG2 cell line, which has been previously shown by our lab to respond to growth-inhibitory AhR ligands. HepG2 cells were plated in microtiter plates, grown overnight, and treated with SU5416 at 40 or 20 μM for 72 hours, at which time they were assayed using the Glo-Titer reagent. The results revealed that HepG2 cells were strongly growth inhibited by SU5416 at both 20 and 40 μM (Figure 6J). The difference between the Glo-Titer signals for vehicle and SU5416 treated cells were statistically significant (Vehicle vs 40 μM SU5416, *p* < 0.0001; Vehicle vs 20 μM SU5416, *p* < 0.0001, and 40 vs 20 μM SU5416, *p* < 0.05). This result suggested that SU5416 had a growth inhibitory effect on HepG2 cells similar to Hepa1 cells. In addition, we performed flow-cytometric analysis of 72 hour treated HepG2 cells, and noted that the forward scatter parameter, which we took as an indicator of approximate cell volume, was 34% and 39% larger than the value for vehicle treated cells with 20 μM and 40 μM SU5416 (data not shown).

## DISCUSSION

Development of the aryl hydrocarbon receptor as a viable target for clinical drugs, especially for cancer and autoimmune diseases, hinges on the identification of ligands with significant AhR-dependent effects in model systems. Potential small-molecules targeting the AhR should selectively modulate the receptor to facilitate a desirable biological/clinical endpoint while avoiding the spectrum of proposed toxic affects attributed to certain classes of AhR ligands, notably dioxins. A logical place to begin the identification of such selective AhR modulators is with existing clinical drugs, several of which are known to activate the receptor to potentially mediate disease-relevant endpoints. In line with this effort, we recently showed that the clinically used drugs leflunomide (Arava^TM^), used for the treatment of rheumatoid arthritis, and the selective estrogen receptor modulator raloxifene (Evista^TM^), used for the treatment of osteoporosis and breast cancer chemoprevention, inhibit the growth of melanoma and estrogen-receptor negative cells, respectively, in an AhR-dependent manner [12, 23, 27].

In order to identify new AhR ligands, we employed a small molecule library screening strategy, which led us to the identification of SU5416/Semaxanib, a potent, selective inhibitor of the VEGF receptor Flk-1/KDR [14] that has progressed to testing in a number of clinical trials. Phase I dose escalation studies of SU5416 for the treatment of solid tumors, either alone or in combination with other chemotherapeutic agents, indicate that SU5416 is well tolerated [17, 18]. However, SU5416 proved ineffective at inhibiting various cancer types, including metastatic colorectal cancer, advanced renal cell carcinoma, melanoma, and soft tissue sarcoma [15, 33, 34]. Despite SU5416′s failure to progress to FDA-approved drug status, it continues to be studied as a potential anti-cancer agent and probe compound for evaluating the effectiveness of VEGFR inhibition strategies for the treatment of cancer. The therapeutic effects of SU5416 in hepatocellular carcinoma have not been tested.

We observed significant activation of the AhR by SU5416 (Figure 1). Furthermore, re-analysis of an *in vivo* SU5416 experiment in rats revealed increased expression of an AhR-gene signature (Figure 2) [24]. One of the exciting aspects of this discovery was that the tissue analyzed for this experiment was collected more than three weeks after a single injection of 20 mg/kg SU5416, suggesting sustained activation of the receptor. That such continued activation of AhR signaling can be achieved at physiologically relevant doses in the absence of side effects is striking, and supports continued investigation of SU5416 as an AhR-targeted drug. Limited-proteolysis and EMSA assays (Figure 2) strongly support the possibility that SU5416 directly binds the AhR.

Due to our interest in developing AhR-targeted anti-cancer agents, we were intrigued by the anti-proliferative effects of SU5416 in hepatoma cells. Proliferation assays as well as real-time monitoring of proliferation showed that SU5416 potently inhibited cellular proliferation of Hepa1 cells while also stimulating a significant alteration of Hepa1 morphology (Figure 3E). Using TAO cells with reduced AhR expression, we found that the AhR was required for mediating the anti-proliferative effects of SU5416. Consistently, we found that Arnt was required for the effects of SU5416 in hepatoma cells, suggesting that SU5416 mediates its effects in Hepa1 cells via transcriptional activation of the AhR. Based on our observation of inhibition of proliferation and G1 arrest in Hepa1 cells and data in the literature, we hypothesized that either p21^waf1/cip1^ and/or p27^kip1^ may be a likely target of SU5416 mediated AhR activation. Expression of p21^waf1/cip1^, but not p27^kip1^, was significantly upregulated by SU5416 (Figure 6), suggesting that this compound may selectively modulate the AhR. During these experiments, we also noted that TCDD failed to induce p27^kip1^ in hepa1 cells. Previously, TCDD has been shown to induce p27^kip1^ at the level of transcription in 5L hepatoma cells [4]. We attributed the lack of p27^kip1^. We demonstrated that upregulation of p21^waf1/cip1^ by SU5416 required both the AhR and Arnt (Figure 6E–6F). Importantly, we also showed that SU5416 was able to facilitate AhR recruitment to the p21 promoter as confirmed by chromatin-immunoprecipitation (ChIP) experiments (Figure 6I).

SU5416 inhibits SH-SY57 and SK-N-BE2 neuroblastoma cell proliferation, which is potentiated by EGCG [35]. SU5416 also increases the cytoxicity of cisplatin in ovarian cancer cells [36]. It is not known if the AhR also mediates the effect of SU5416 in these cells. Interestingly, SU5416 induces G1 arrest and increases expression of p21^waf1/cip1^ in late outgrowth endothelial cells isolated from patients with neovascular age-related macular degeneration at low to mid-micromolar concentrations [37]. Pharmacokinetic analysis of SU5416 indicates it is rapidly cleared from the plasma, primarily via hepatic metabolism, with half-lives for elimination of the parent compound ranging from approximately 30 minutes to >1 hour, and which do not vary dramatically across different species and doses of SU5416 [38]. The rapid clearance of SU5416 in the context of its effectiveness in inhibiting tumors in mouse xenograft models despite only biweekly treatments (25 and 50 mg/kg) has been investigated [39]. SU5416 was shown to be rapidly taken up by human umbilical vein endothelial cells (HUVECs), with exposure to 4 μM [^14^C]-SU5416 in cell culture media for 3 hours resulting in a cellular concentration of 450 μM, which decreased to ∼25 μM following a washout period [39]. Interestingly, the concentration of SU5416 in HUVECs after the washout period was stable for more than 48 hours [39]. These results are consistent with our identification of an AhR-activation signature in rat lungs several weeks after administration of a single dose of SU5416 (Figure 2).

The high concentrations of SU5416 that can be achieved *in vivo* in a clinical setting are of significant interest, especially in the context of a persistent bioaccumulation of SU5416 in cells in culture [39] and *in vivo* as well (Figure 2). Taken together, the observations that SU5416 is relatively well tolerated (∼145 mg/m^2^ 1–2 doses/weeks) in patients, significantly activates the AhR *in vivo* (Figure 3), and has significant anti-proliferative activity that requires AhR signaling (Figures 3, 4, 5) strongly supports the possibility of developing SU5416 as an AhR-based cancer therapeutic. As *in vivo* C_max_ levels of SU5416 can reach approximately 30 μM after intravenous administration at a dose of 145 mg/m^2^ [18], the prospects of achieving sustained AhR activation in humans are encouraging.

Our study provides support for development of AhR-based therapeutics against cancer, especially hepatocellular carcinoma where the AhR is highly expressed. The functional differences in AhR activation by TCDD and other AhR ligands [11, 27, 40–43] remains an important topic that continues to be investigated. Possibilities include modulation of the AhR in a ligand-selective manner to interact with distinct DNA-binding sequences, recruitment of new co-activator proteins, or induction of complementary cellular pathways that act in conjunction with the AhR signaling that result in AhR ligand-specific phenotypes. In studies using in an *in vitro* random oligomer enrichment assay with six unique AhR activating chemicals, binding of the AhR to the core XRE sequence is not dramatically altered by these ligands [43]. Another study compared differential gene expression elicited by TCDD, PCB126, βNF, or ICZ in mouse hepatoma Hepa1c1c7 cells and C57BL/6 mouse liver cells, and reported that only 35% of differentially expressed genes induced by these four ligands in Hepa1c1c7 cells are common, arguing for selective modulation of the receptor [44].

In conclusion, we characterized a novel anti-proliferative effect of SU5416 in hepatoma cells that strongly requires the expression of AhR. Upregulation of p21^waf1/cip1^ by SU5416 may be responsible for the observed anti-proliferative effects of SU5416 in hepatoma cells, and potentially other cell types as well.

## MATERIALS AND METHODS

### Reagents, cell culture, and reporter gene assays

Acquisition, verification, and culturing conditions of Hepa1, TAO, C4, vT{2}, and HepG2 cells have been described previously [23]. STR DNA profiling (Genetica cell line testing) was used to confirm the human HepG2 cell line. Cells were cultured in DMEM supplemented with 10% FBS (Tissue Culture Biologicals, CA USA) and a solution of penicillin and streptomycin (Cellgro, USA) and grown in a 5% CO_2_ humidified incubator. Cells were routinely passaged every 2–3 days. SU5416 (purity ∼99.8%, HPLC) was purchased from Tocris Bioscience. Reporter gene assays were conducted as described previously [23].

### Western blotting and immunofluorescence

Immunofluorescence staining for determining cellular localization of the AhR was performed as described previously [45]. Western blotting was performed as described previously [46]. The p21 antibody was from BD Biosciences and was used at a dilution of 1:400.

### Limited AhR proteolysis assay and EMSA

Limited AhR Proteolysis assays were performed as described previously with modification [47]. The source of AhR was from whole cell extracts of Hepa1 cells. Briefly, Hepa1 extracts (48 μg) were incubated with DMSO, TCDD, or SU5416 at the indicated concentrations for 1 hr and proteolyzed by 1 μg/ml subtilisin for 1, 2, 4, or 8 min. Reactions were quenched by the addition of SDS-containing sample buffer. The resulting protease cleavage fragments were separated by SDS-PAGE and visualized by Western blotting using a polyclonal AhR antibody (Enzo Life Sciences) that recognizes the N-terminus of the AhR. Electrophoretic mobility-shift assays (EMSA) were performed as described previously [12].

### Chromatin immunoprecipitation

To determine if the AhR protein binds to the mouse p21 gene, we carried out chromatin immunoprecipitation according to a published procedure [48]. Briefly, Hepa1 cells were treated for 12 h with 20 μM SU5416 or 0.1% DMSO. The cells were incubated for 10 min with 1% PFA followed by 5 min incubation in 0.125 M glycine. Then, cells were rinsed twice with cold PBS and trypsinized. The cell pellet was resuspended in cell lysis buffer (5 mM PIPES, pH 8.0, 85 mM KCl, 0.5% NP-40, 1x protease inhibitor cocktail) and the nuclei was pelleted by centrifuging for 5 min at 5,000 rpm at 4°C. The nuclei pellet was resuspended in nuclei lysis buffer (50 mM Tris-Cl, pH 8.0, 10 mM EDTA, 1% SDS, 1x protease inhibitor cocktail), and sonicated to obtain fragmented chromatin. One microgram of AhR antibody (Biomol, BML-SA-210) or heat-inactivated non-specific antibody was added to the chromatin and incubated overnight at 4°C on a rotating platform. Pre-blocked protein A/G Plus agarose (SantaCruz) with BSA and salmon sperm DNA was added to the immunocomplex in the presence of BSA and salmon sperm DNA and incubated for 2 h at 4°C. The agarose beads were washed twice with wash buffer-I (50 mM Tris-Cl, pH 8.0, 2 mM EDTA, 10 μg/ml PMSF) and four times with wash buffer-II (100 mM Tris-Cl, pH 8.0, 500 mM LiCl, 1% NP-40, 1% sodium deoxycholate, 1 μg/ml PMSF), and eluted with elution buffer (50 mM sodium bicarbonate, 1% SDS). The cross-linkage was reversed by incubating the solution for 5 h at 65°C in the presence of 33 μg/ml RNase A, and the DNA was purified by using PCR purification kit (Qiagen). The purified DNA was used as a template for PCR, which was performed according to standard procedures using primers described by Schnekenburger *et al*. and Jackson *et al*. for CYP1A1 and p21, respectively [30, 49].

### Real-time PCR

Collection of RNA, generation of cDNA, and qPCR assays were performed as described previously [12, 23]. qPCR primers for mouse CYP1A1, p21, p27, and GAPDH were as follows: CYP1A1: FP 5′- GACCCTTACAAGTA TTTGGTCGT-3′, RP 5′-GGTATCCAGAGCCAGTAACCT-3′; p21: FP 5′- CCTGGTGATGTCCGACCTG-3′, RP 5′-CCATGA GCGCATCGCAATC-3′; and p27: FP 5′-TCAAACGTGA GAGTGTCTAAC G-3′, RP 5′-CCGGGCCGAAGAGATTT CTG-3′; and GAPDH: FP 5′-AGGTCGGTGTGAACGG ATTTG-3′, RP 5′-TGTAGACCATGTAGTTGAGGTCA-3′. Primers for human CYP1A1 and GAPDH were as described previously [12].

### Cell image analysis and flow cytometry

Images of cells at 10× magnification were obtained with an inverted Zeiss microscope attached to a video camera using Image J software. Processing of images to determine number of cells and cell density was performed using ImageJ software (NIH). The number of cells per field was counted using the cell counter function, in which visible nuclei were recorded as one cell. The area of each image occupied by cells was determined by a threshold function, in which the contrast of cells against the culture plate was adjusted until the former was highlighted as ‘black’ and the area without cells was white. The respective areas of black and white were calculated, and the total cell area was divided by the number of cells to give an approximate mean cell area. The mean cell areas determined for each image were then averaged and compared using ANOVA.

Cell cycle analysis was performed by methanol fixation of cells followed by incubation with propidium iodide and RNase A. Annexin V and CFSE staining was performed as described previously [31]. Flow cytometry for cell cycle analysis and apoptosis/proliferation assays were performed with Beckman Coulter FC500 and CYTOFLEX S instruments, respectively. Data were analyzed with FlowJo (Version 10.2, TreeStar Inc., USA).

### Proliferation assays

Determination of cell proliferation and real-time monitoring of cellular growth was performed as described previously using CellTiter-GLO substrate (Promega, USA) and XCelligence (Roche, USA), respectively [12]. Colony forming assays were performed as described previously with modification [43]. Briefly, cells were seeded at a density of 250 cells/plate in 60 mm dishes, grown overnight, and then treated with compounds by replacement of cell culture media. Treatment duration was either for a 24-hour pulse, after which the media containing compounds was removed and replaced with fresh media, or left for continuous treatment.

### Data analysis

Microarray data files were obtained from the Gene expression omnibus (GEO Accession# GSE8134). Data were processed with R as described previously [24]. All other data were analyzed by one-way ANOVA with the Tukey multiple comparison test using Prism software (Version 6.0a, Graphpad Software, La Jolla, CA). *P values* less than 0.05 were considered statistically significant.

## SUPPLEMENTARY MATERIALS FIGURES


