# Supplementary material for: The aryl hydrocarbon receptor is required for induction of p21cip1/waf1 expression and growth inhibition by SU5416 in hepatoma cells
Source: Oncotarget. 2017 Mar 9;8(15):25211–25. doi: 10.18632/oncotarget.16056 (PMC5421923; doi:10.18632/oncotarget.16056)
Supplement: Supplementary file 1 [file oncotarget-08-25211-s001.pdf]

The aryl hydrocarbon receptor is required for induction of p21[cip1/waf1] expression and growth inhibition by SU5416 in hepatoma cells

Supplementary Materials

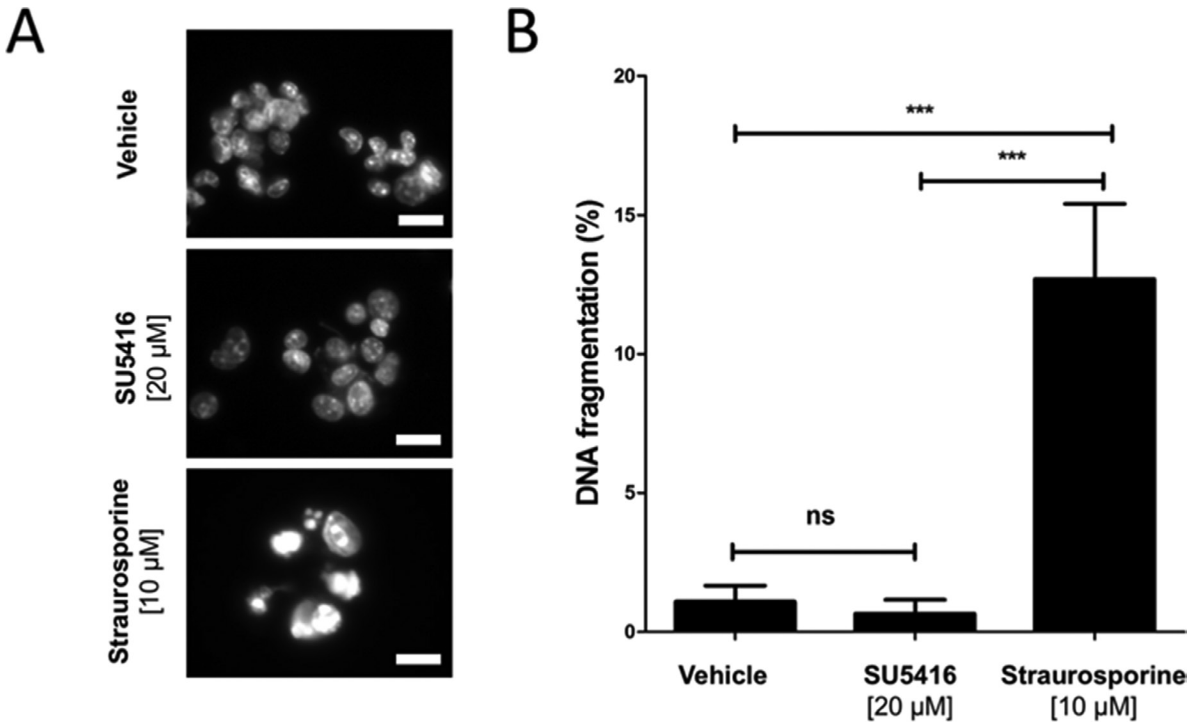

Supplementary Figure 1: SU5416 does not induce apoptosis. (**A**) Hepa1 cells treated with vehicle, SU5416 20 µM or Staurosporine 10 µM (as a positive control) for 72 hours. Cells were stained using DAPI and analysed for DNA fragmentation by fluorescent microscopy. (**B**) Quantitation of DAPI staining results. % DNA fragmentation indicates the percentage of cells with evidence of nuclear fragmentation. At least 300 cells were assessed per treatment group. (***< 0.0005, Tukey analysis following ANOVA).
